# Investigation on the isotopic exchange radiofluorination of the pentafluorosulfanyl group

**DOI:** 10.1039/d5ob01419k

**Published:** 2025-10-29

**Authors:** Hugh Hiscocks, Gabrielle Weerasinghe, Weicong Huang, Fabio Colasuonno, James Hill, Patrick Ryan, Rhys Johnston, Allen Brooks, Peter J. H. Scott, Alison Ung, Martina Lessio, Luke Hunter, Giancarlo Pascali

**Affiliations:** a School of Mathematical and Physical Sciences, University of Technology Sydney Ultimo 2007 NSW Australia; b School of Chemistry, University of New South Wales Kensington 2033 NSW Australia; c Institute for Molecular Bioscience, The University of Queensland Brisbane Queensland 4072 Australia; d Department of Radiology, University of Michigan Ann Arbor Michigan 48109 USA; e Department of Medicinal Chemistry, The University of Michigan Ann Arbor Michigan 48109 USA; f Department of Pharmacology, The University of Michigan Ann Arbor Michigan 48109 USA; g Australian Nuclear and Science Technology Organisation, Lucas Heights 2234 NSW Australia gianp@ansto.gov.au; h Brain and Mind Centre, University of Sydney Camperdown 2050 NSW Australia

## Abstract

The pentafluorosulfanyl group (–SF_5_) is one of the most promising fluorinated functional groups, recently developed as an alternative to the trifluoromethyl group (–CF_3_) in drug design. Fluorine-18 allows researchers to investigate *in vivo* activity and biodistribution of novel fluorinated drugs; however, currently no methods are reported to radiolabel –SF_5_ moieties. In this work we report the first successful radiolabelling of such a group by isotopic exchange, and we show peculiar reaction trends. We studied this reaction using model compounds and functionalized amino acids, also adopting an unbiased approach to reaction optimization to minimize cognition bias. The results have been analyzed by standard statistical methods and Artificial Intelligence (AI) tools. Finally, we serendipitously discovered the production of two radioactive products from one precursor, that we hypothesize being positional radioisotopologues that interact differently with the chromatographic stationary phase; if further proven, this finding hints, for the first time, at a case of chemical differences between molecules containing ^19^F and ^18^F.

## Abstract 2

This study reports the first radiofluorination of pentafluorosulfanyl groups *via *isotopic exchange, analyzes reaction conditions with AI and statistical tools, and proposes the formation of positional isotopologues.

## Graphical abstract

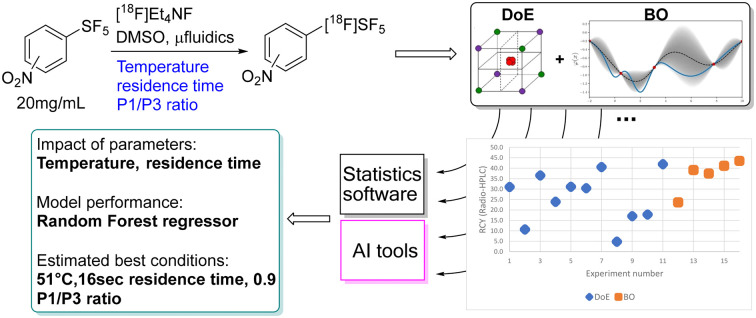

## Introduction

The pentafluorosulfanyl functional group (SF_5_) is gaining interest as a substituent in the design of novel drug leads.^1,2^ Its octahedral geometry around the sulfur center provides a chemical environment that imparts unique physical properties. This group is typically compared with the more established CF_3_ functional group, of which it represents a potential alternative in the design of new molecules. SF_5_ has a higher electronegativity and dipole moment than CF_3_, but contemporarily it also exhibits increased lipophilicity, thus being dubbed as “super-CF_3_”.^3^ In addition, it has a steric hindrance bigger than CF_3_ but smaller than *t*-Bu. This moiety is extremely stable to hydrolysis and is promising for *in vivo* applications of related drug leads.

Therefore, several researchers are investigating methods to specifically introduce SF_5_ groups into relevant structures,^1,4–8^ including privileged scaffolds for developing new drug leads.^9–15^ The native presence of fluorine atoms suggests the opportunity of achieving ^18^F-labelling of the moiety. Fluorine-18 is a positron-emitting radionuclide readily available from small medical cyclotrons that has excellent imaging properties and a half-life (110 min) suitable for distribution,^16^ paving the way to using Positron Emission Tomography (PET)^17^ to investigate relevant pharmacological parameters of SF_5_-functionalized drugs, similarly to prior efforts with the CF_3_, PF_5_ and BF_3_ group.^18–20^ However, no radiolabelling methods are available to date for SF_5_. In this work, we present the first examples of ^18^F-labelled SF_5_ molecules, obtained by isotopic exchange under both vial and flow microfluidic environments. We discuss our peculiar findings on substrate scope, reaction conditions and product identity, employing Artificial Intelligence (AI) tools to conduct data analysis.

## Results and discussion

### Radiolabelling of commercial model substrates

**Fig. 1:**
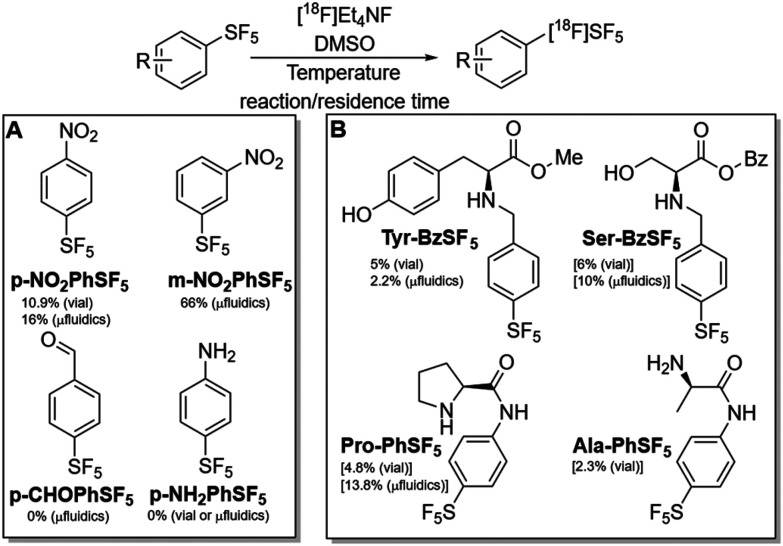
Radiolabelling precursors used in this study and respective radiochemical conversions (RCC) obtained; commercially available aromatics containing SF_5_ (panel A) and synthesized amino acid analogues (panel B). Best RCC values are reported with indication of the reaction modalities used; values in square brackets refers to the RCC for the unknown radioactive product.

We focused our initial investigation on commercially available model aromatic compounds, bearing both the SF_5_ and another functional group (Fig. 1A), to gauge the effect of substituents on the exchange radiolabelling reaction. We conducted labelling using both microfluidic (SI1) and vial approaches employing azeotropically dried [^18^F]Et_4_NF as the radiolabelling species (obtained from Et_4_NHCO_3_). In this regard, the use of a flow microfluidic system allowed us to test efficiently a wide range of temperatures, in line with previously published optimization procedures.^21^ In addition, we tested these reactions on different days and laboratories, employing the reaction and analytical systems available at each site; while this approach produced results that are not absolutely comparable, this still allowed the identification of a few key common trends and showcased the generalizability of the radiochemistry.

**Fig. 2:**
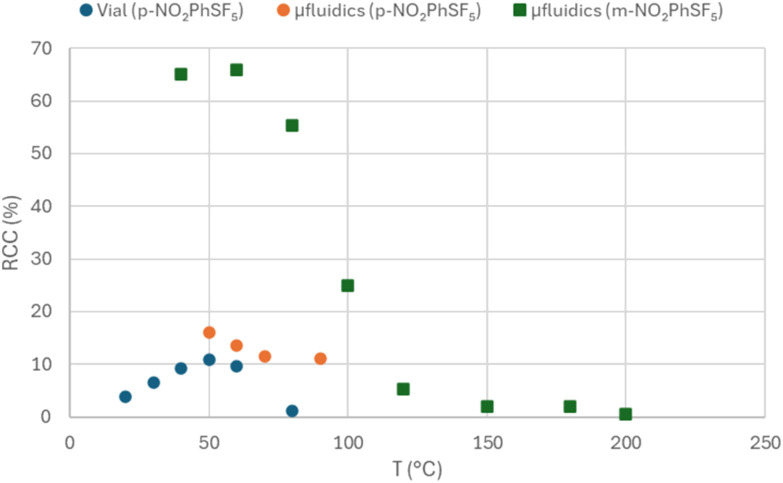
Relation of RCC with reaction temperatures, for radiofluorination reactions performed in vial and microfluidics environments in analogue conditions (10 mg mL^−1^ precursor concentration, DMSO solvent, [^18^F]Et_4_NF reagent, *n* = 1).

Of the four precursors tested, only the ones bearing a nitro substituent provided a detectable radiochemical conversion (RCC, Fig. 2), with *m*-NO_2_PhSF_5_ affording a higher yield (maximum RCC of 66 *vs.* 16% for *p*-NO_2_PhSF_5_). The use of dried radiofluorination complex was required to achieve such conversions; we tested using non-dried solution, and this modification did not afford any isotopic exchange. Given that the aldehyde-substituted precursor did not provide any RCC, it is unlikely that the electron-withdrawing effect of the substituents on the aromatic ring plays an important role in this exchange reaction. Additionally, it was verified that the highest RCC can be obtained by heating the reaction in the 40–60 °C range; lower and, surprisingly, higher temperatures are detrimental to yield. Both aspects suggest that this radiofluorination process does not follow the conventional mechanisms of nucleophilic substitution reactions. When comparing the results obtained with *p*-NO_2_PhSF_5_ in vial and microfluidic systems, it is evident that the latter option provides slightly better yields (maximum RCC of 16 *vs.* 10.9%) but, most importantly, uses a markedly reduced amount of precursor and reaction (*i.e.* residence) time of 23 s *vs.* 20 min.

Given that the highest yields were achieved at lower temperatures, we investigated the stability of the product in the reaction mixture over time, by reanalyzing it after 4 h of ageing at room temperature. Additionally, we analyzed the mixture after adding water as a quenching agent, and tested the time stability of this diluted sample. We performed these stability assessments in triplicate on [^18^F]*m*-NO_2_PhSF_5_ reaction mixture, using the best microfluidic radiolabelling conditions. Room temperature ageing for 4 h provided a 22 ± 4% reduction in RCC; on the other hand, we did not see a significant reduction upon water quenching (6 ± 10% RCC reduction) or after its 4 h ageing period (7 ± 8% reduction). A shorter stability test of the unquenched reaction mixture was also performed, and demonstrated a reduction of RCC of 3 ± 2% at 30 min and 7 ± 3% at 60 min. These results suggest that some level of radioisotopic scrambling, possibly due to adventitious ^19^F in the mixture, still occurs at room temperature, and that water quenches this phenomenon effectively.

**Fig. 3:**
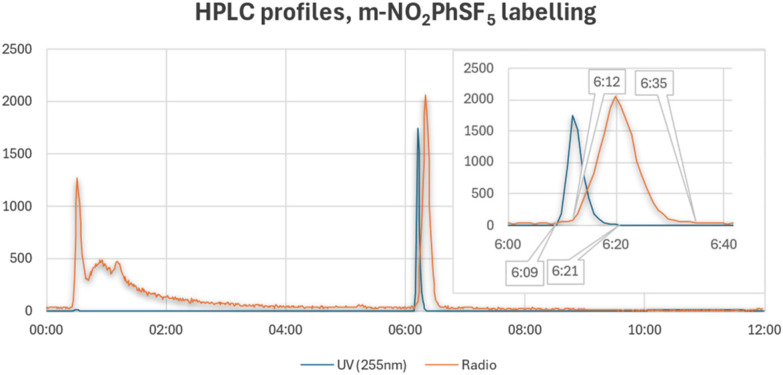
Example of HPLC profiles for radiolabelling reaction of *m*-NO_2_PhSF_5_, evidencing the different width of UV (blue) and radio (orange) peaks (CH_3_CN (A), H_2_O (B) with 0.05% TFA; 2 mL min^−1^; (A) 5% 0–2 min, 5–95% 2–12 min, 95–5% 12–13 min, 5% 13–18 min; Chromolith® RP-18 50 × 4.6 mm).

Finally, we observed that the radio-HPLC profiles of these reaction mixtures showed radioactive peaks that were markedly wider than their stable isotopologues (20–40 s *vs.* 10–12 s), in the worst cases hinting at the potential of an unseparated double peak (Fig. 3, SI2). While this could be due to injected activities, detector sensitivity and detection loop, we did not experience such noticeable differences with other compounds injected in the same system under analogous conditions, especially when using a gradient method with 2 mL min^−1^ eluent flow rate, as in these analyses. However, at this stage we attributed these peaks to the one product, guided by previous experience with broad peaks noted in other chromatographic systems or projects.

Exchange radiofluorination was also tested on *m*-NO_2_PhSF_5_ employing [^18^F]ethenesulfonyl fluoride (ESF) as labelling reagent and Et_4_NHCO_3_ as additive,^22^ and afforded 32 ± 1% and 43% RCC in a vial environment for 15 min at 100 °C and 60 °C respectively. This reaction revealed an unprecedented transfer of ^18^F among two different sulfur centers, clearly highlighting the different stability of S–F bonds in sulfonyl and pentafluoro sulfanyl groups.

### Radiolabelling of SF_5_-functionalized model amino acids

In order to investigate this exchange radiofluorination on more complex substrates, we synthesized protected amino acids functionalized with SF_5_-containing arenes (Fig. 1B), following recently published protocols.^23–25^

**Fig. 4:**
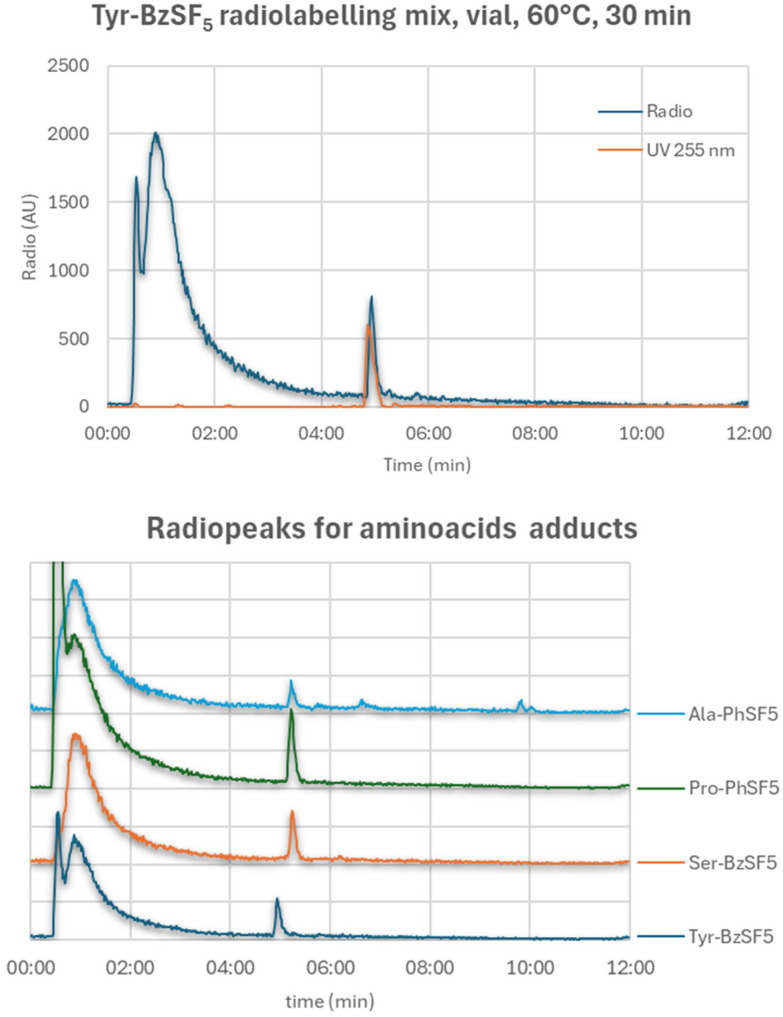
(top) HPLC profile for the exchange radiofluorination of Tyr-BzSF5, evidencing the *R*_t_ alignment of precursor and radio product; (bottom) HPLC profiles of the radioactive trace for the exchange radiofluorination of the 4 amino acid adducts, evidencing the same *R*_t_ for all the radiopeaks, except for the Tyr adduct. (CH_3_CN (A), H_2_O (B) with 0.05% TFA; 2 mL min^−1^; (A) 5% 0–2 min, 5–95% 2–12 min, 95–5% 12–13 min, 5% 13–18 min; Onyx C18 50 × 4.6 mm).

These reactions were tested in a vial and microfluidic environment (SI1), and reaction mixtures analyzed by radio-HPLC (SI3). We found out that only Tyr-BzSF_5_ afforded the desired product, although in a low RCC (Table 1 and Fig. 4, top); however, some reaction mixtures for this precursor revealed the presence of a later eluting peak that seemed to be common, by HPLC retention time, to the only radioproduct that was detected with all the other precursors. For all these radioactive products, higher temperatures provided lower RCC, although the best values were achieved at temperatures tendentially higher than the best ones found for the nitrobenzene model precursors.

**Table 1:** Synoptic table of exchange radiofluorination reactions on functionalized amino acids, highlighting the only radioproduct with confirmed identity

Precursor	Vial^a^: RCC^b^@temperature	mfl^c^: best RCC^b^@temperature	UV peak^d^*R*_t_ (min)	Radiopeak *R*_t_ (min)
Tyr-BzSF_5_	4.8%@60 °C	2.2%@50 °C	4 : 48	4 : 56
	5%@100 °C			
Ser-BzSF_5_	(6%@60 °C)	(10%@70 °C)	5 : 12	(5 : 14)
	(5%@100 °C)			
Pro-PhSF_5_	(4.8%@60 °C)	(13.8%@110 °C)	6 : 33	(5 : 13)
	(2.1%@100 °C)			
Ala-PhSF_5_	(2.3%@60 °C)	NA	5 : 37	(5 : 13)
	(2.3%@100 °C)			

a 1 mL of 20 mg mL^−1^ in DMSO added with 0.1 mL of radiofluorination dried complex, 30 min reaction time.

b Unknown radiopeak data in parentheses.

c 10 mg mL^−1^ reaction bolus concentration in DMSO, 23 s residence time, temperature giving highest RCC reported.

d *R*_t_ for the precusor isotopologue; the *R*_t_ gap between radio and UV is +4–8 s with the used flow rate.

The identity of the common radiopeak detected in these reactions remains unknown (Fig. 4), and it is potentially due to the radiolabelling of a common SF_5_-containing fragment generated under the reaction conditions. We believe the fact that RCC is reduced at higher temperatures indicates the peculiar isotopic exchange behaviour at the SF_5_ site. In addition, given the high concentrations (>10 mg mL^−1^) required to achieve detectable RCC, and the absence of precursor degradation products in the UV trace, it is unlikely that such radiopeaks are due to the radiofluorination of impurities originally present in the synthetic substrates.

In the case of Ser-BzSF5, the attribution remains uncertain, as the UV peak of the precursor is extremely close to the HPLC *R*_t_ of the unknown radioproduct. These results support the potential for direct radiolabelling of amino acids or peptides preliminarily functionalized with SF_5_-arenes. However, further studies are needed to increase the RCC, *e.g.* by understanding the nature of the common radioactive peak, or by identifying improved conditions, arene substituents’ pattern or useful catalysts. On the other hand, the higher yielding access to radiolabelled nitroarenes suggests that an indirect peptide labelling strategy could be more feasible, involving the reduction of the nitro intermediate and successive reductive amination or amide coupling to the core of the desired probe.

### Reaction optimization strategy

This peculiar reactivity motivated us to further explore the chemical space compatible with this new chemistry, prompting us to employ an unbiased reaction optimization strategy to gauge the impact of reaction parameters, while minimizing cognition bias and experimental effort. Such an approach was intended to minimize the number of experiments required, thus alleviating the reduced access to radiofluorination facilities caused by the closing of our cyclotron site, also following financial repercussions of the 2019–2021 pandemic situation.^26^

For this campaign, we re-installed an Advion microfluidic system into an external radiochemistry laboratory, already equipped with a radio-HPLC system analogue to the one previously used, but mounting a different type of monolith column. We chose to only use a microfluidic approach, given the capacity to run multiple radiolabelling tests per day using the same starting radiofluoride amount.

**Fig. 5:**
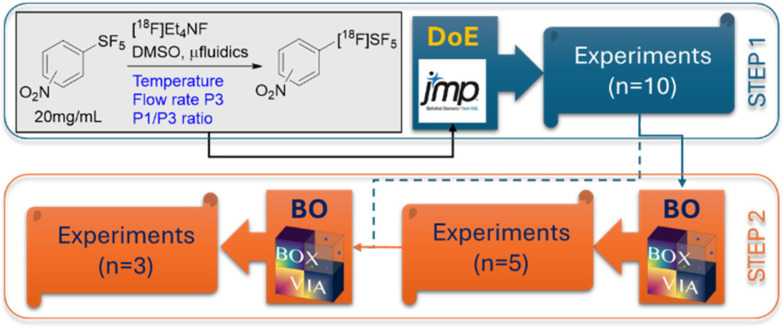
Exploration/optimization strategy used in this study, highlighting the two key procedural steps of design-of-experiment (DoE) and bayesian optimization (BO).

Our strategy was based on a two-step approach (Fig. 5) and was applied to *m*-NO_2_PhSF_5_ and *p*-NO_2_PhSF_5_. In the first step, we built a Design-of-Experiment (DoE) campaign of 10 experiments for each precursor (SI4),^27,28^ designed using JMP© software,^29^ modifying key reaction parameters: temperature (range: 30–90 °C), radiofluorinating complex flow rate (*i.e.* P3 flow rate, range: 10–50 μL min^−1^) and precursor/radiofluoride volume ratio (*i.e.* P1/P3 ratio, range: 0.5–2). In the second step, we used a Bayesian Optimization (BO) freeware application (*i.e.* BOXVIA),^30^ using the DoE results as a training set, to propose the subsequent 5 experiments to approximate the best conditions. Once completed, we added these new results to the training set and repeated the algorithm to forecast the final 3 experiments. In order to minimize precursor consumption, we fixed the P1/P3 ratio to 1 in the BO runs. Apart from reducing the number of runs to identify (5 *vs.* 3), the 1^st^ BO was set to facilitate exploration of new conditions, while the 2^nd^ BO was set to prefer exploitation of already acquired results. While this approach may be limiting compared with other examples in the chemical reaction space,^31–33^ our choice was motivated by the reduced availability of resources and the potential for such a workflow to be realized in a single experimental day with the same starting radioactivity.

**Fig. 6:**
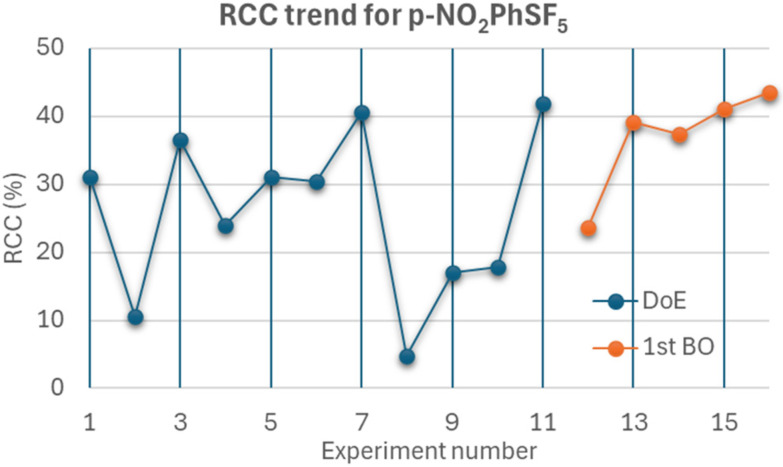
RCC values for the exchange radiofluorination of *p*-NO_2_PhSF_5_ recorded after the DoE and 1^st^ BO set of runs, evidencing the improvements provided by the optimization strategy.

Due to constraints on facility access, the limitations of this study prevented us from completing the entire optimization process in one day, and the recorded values of RCC were not directly comparable across different days. Therefore, it was not always straightforward to directly visualize the performance of our optimization strategy and easily identify the best reaction conditions; however, we were able to perform the DoE and the first BO runs on the same day on one instance, and we noticed a clear improvement in RCC (Fig. 6), thus demonstrating the future validity of such a strategy in well-controlled situations.

### Statistical approaches to analyze reaction data

Given the heterogeneity of conditions and results across the various experimental campaigns, we decided to consolidate the microfluidic reaction data for the two nitro-precursors (although we excluded runs with evident instrumental issues, leading to 75 total entries, SI5) and to perform a statistical analysis to gauge the impact of radiolabelling parameters and hence verify the peculiar trends for this isotopic exchange process. To further assess the best workflow for future applications, we used the statistical toolbox available in Microsoft Excel, JMP©^34^ and compared their results, scope and usability with Julius© AI.^35^ In order to propose a workflow that could be adopted by a wider group of radiochemists, we focused on identifying the analysis method that was the simplest to perform, only required basic training and statistical knowledge, and whose outputs were most straightforward to interpret.

Using the Microsoft Excel toolbox, we calculated the Pearson correlation factors (SI6) and focused on the correlation with RCC. We found a slight positive correlation with the amount of precursor (0.238), total bolus size (0.193) and P1/P3 ratio (0.179); we interpreted this outcome as three different ways to express the same concept: the RCC is higher when a higher absolute amount of precursor is employed, that is frequently the case with exchange radiofluorination processes.^36^ Stronger negative correlations were found with temperature (−0.547), “*m*- *vs. p*-” (−0.441) and residence time (−0.319); these correlations highlighted how lower temperature and shorter residence times resulted in higher RCCs. It is worth noting that non-numerical (*i.e.* categorical) parameters, such as “*m*- *vs. p*-” or “Quenching”, had to be translated into numbers for Excel to perform tests on them. For this reason, the strong negative correlation on “*m*- *vs. p*-” indicates that the *m*-precursor gave higher RCCs, as it was given a value of “1”, while the *p*-compound was given “2”.

The same toolbox allowed calculation of the covariance factors with RCC (SI7), which further supported the correlation results as a large positive value was found for the precursor amount (4508.8), while a substantial negative covariance was found for reaction temperature (−383.5).

We did not use other statistical tests in Excel, as the two we performed were cumbersome to create and yielded fairly static results (*e.g.* modifying the analysis conditions slightly required repeating all the steps).

The JMP© package^34^ provides many preset statistical tools to further understand data trends and impact. Understanding how to use each of these tools requires extensive training and statistical knowledge. Although it would be useful to expert users, we selected some of the simpler tests that are readily performed with just introductory knowledge. First, we confirmed the alignment with the Excel toolbox results by using the multivariate and principal component (covariance) analysis, whose Pearson correlation and covariance values nearly coincided with the ones previously calculated by Excel (SI8, SI9); the slight differences were probably because we did not translate in numbers the categorical parameters, and therefore these elements were not included in these tests.

JMP© easily provided additional tests; for example, the predictor screening tool allowed us to quickly identify which parameters are most significant in predicting RCC. This analysis calculated that temperature and “*m*- *vs. p*-” substitution had 38.6% and 16.4% contributions to RCC, respectively (SI10). A more detailed analysis of the impact of parameters was available using the response screening tool that confirmed a significant impact of temperature, “*m*- *vs. p*-” substitution and residence time on RCC, while, partly in contrast with simpler analysis, the amount of precursor seemed to have a less significant role (SI11).

Another useful tool from JMP© was the “Fit Least Squares” analysis; however, this calculation required grouping the 4 different categorial combinations. The tool provided useful relational information and, for the largest group (*i.e.* “*m*-” and “not quenched”), it confirmed a strong effect of reaction temperature over the other parameters (SI12). The result window also provided a “Profiler” tool that could be used to identify the conditions giving the highest RCC. However, it required a trial-and-error approach, assuming a linear correlation between parameters and frequently provided sets of parameters that were not attainable in practice.

In order to account for potential non-linear relations between parameters, a neural network response predictor, based on an NTanH model, was also run in JMP©. This tool created parameter relations using the provided data to train the model and validate its trustworthiness. The model achieved a training *R*^2^ of 0.82 and a validation *R*^2^ of 0.65, hence indicating a good capacity to reproduce parameter relations, but potentially suggesting a degree of data overfitting. The “Profiler” tool in this option indicated a maximum RCC of 40–45% attainable at temperatures lower than 90 °C, but using extremely high precursor amounts (*i.e.* >5 mg) and very short residence times (*i.e.* <7 s); these conditions are challenging to realize in the microfluidic system employed in this work (SI13).

It is worth noting that JMP© provided a simpler user experience than Excel, generating results that were easy to tweak (*e.g.* dynamic modifications), as well as useful and interactive graphs. However, it was a comprehensive statistical software with a steep learning curve, requiring significant effort to identify the best tests to perform and interpret their practical meaning.

Having performed this initial investigation and given the growing impact of Artificial Intelligence (AI) in many applications and our field,^37^ we decided to search for an AI solution focused on data analysis and statistics, and we resorted to using Julius© (SI14). After uploading the results table in the interface, we interacted with the AI engine in natural language. First, we asked it to calculate conventional statistic measures (*e.g.* Pearson correlation, covariances), but differentiate between those for *m*- and *p*-precursor, as well as explain their meaning. In doing so, the AI confirmed the trend evidenced by previous analyses, highlighting the substantial impact of temperature and residence time, but providing slightly different values due to the discrimination of the two isomer cases. Generating correlation heatmaps was straightforward, and the results could be modified by reducing the number of parameters to consider. The AI also explained the meaning of the values obtained and found considerable variation of RCC across different dates, thus confirming the importance of realizing this kind of campaign in a well-controlled environment and with time-efficient planning.

**Fig. 7:**
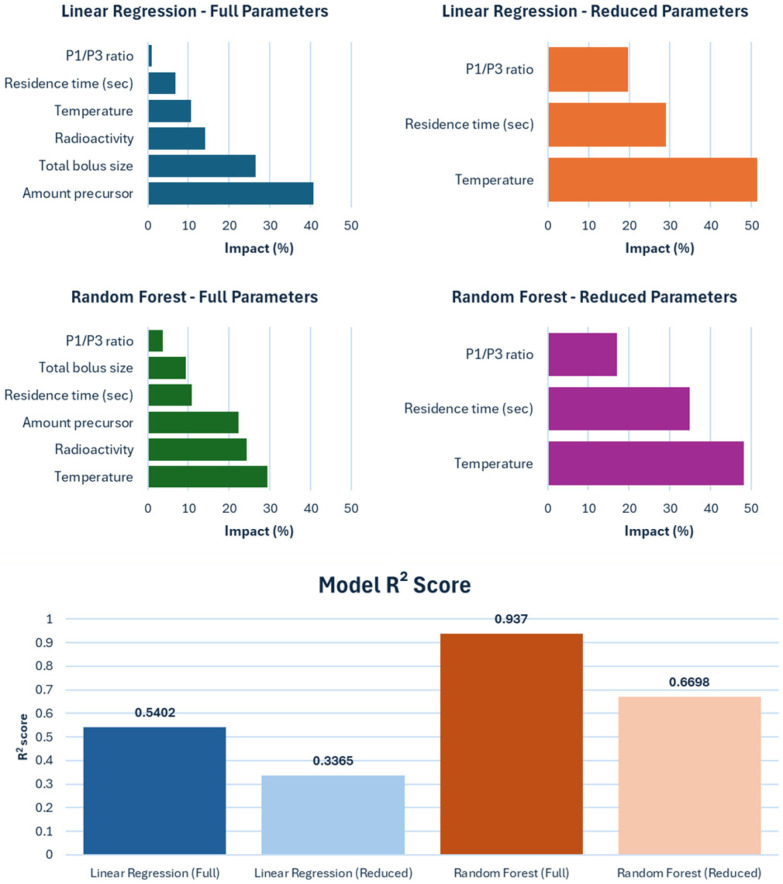
Comparison of percent impact on RCC of parameters, following Linear regression and Random Forest regression analysis (top), and *R*^2^ values for these regressions (bottom), performed by Julius© AI.

We next requested the AI to perform a percentage ranking of parameter impacts using both a metric linear analysis (such as least squares regression) and a random forest regressor, and asked it to provide useful graphs and measures reflecting the trustworthiness of such models (Fig. 7). We asked to run these analyses for both the full set and a reduced set of parameters (*i.e.* Temperature, residence time and P1/P3 ratio, which are the parameters easier to practically modify). These results calculated that the random forest regressor reproduced the reaction parameters’ relations better than the Linear regressor (*R*^2^ of 0.94 *vs.* 0.54 and 0.67 *vs.* 0.34 for, respectively, full and reduced sets) and confirmed that temperature was the most important parameter to improve RCC (29% and 48% impact for, respectively, full and reduced sets), with the amount of radioactivity and precursor having the next strongest influences. Residence time was also found to be impactful (35%) in the reduced set of parameters.

Given the ease of interaction, we then asked which conditions would maximize the RCC, using the 2 models and the 2 parameters sets. Julius© AI's output also differentiated between both precursors (*i.e. m*-/*p*-) and just *m*- or *p*-cases (SI13). The various scenarios were assessed for reliability *via* their *R*^2^ value. It was found that the random forest regressor provided a better fit of the experimental data using the full set of parameters (*i.e. R*^2^ > 0.91) and, to a slightly lesser extent, with the reduced set (*i.e. R*^2^ = 0.67 for *m*-/*p*-case, *R*^2^ = 0.81 for both *m*- and *p*-case). The linear regressor did not give a good representation of the data, thus implying that non-linear relations were present.

**Fig. 8:**
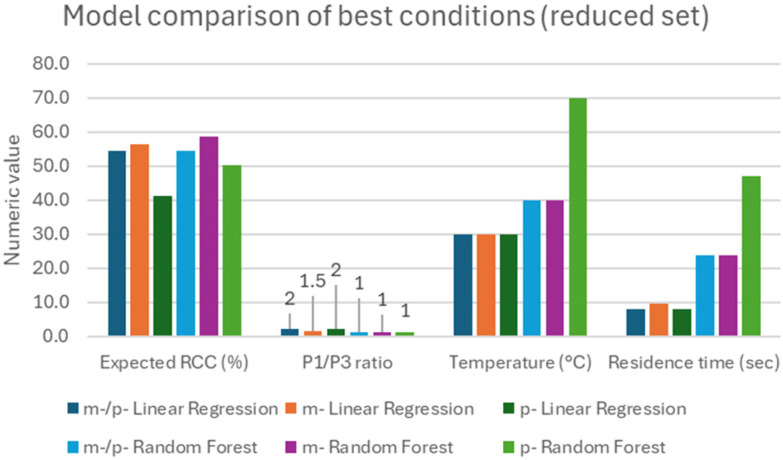
Comparison view of reduced parameter set from the various regressor models for *m*-/*p*-, *m*- and *p*-isomer cases.

The conditions predicted and expected RCC (full data in SI15, extracted data graphed in Fig. 8) were aligned with our radiochemical intuition of the process, whereas lower temperatures and residence times yielded better RCC, with expected values around 50%, and tended to have lower RCC values for the *p*-isomer. On this, it is interesting to notice that the predicted best temperature for the *m*-isomer was lower than the one for the *p*-isomer (40 *vs.* 70–90 °C), which hints at slightly milder conditions required to generate [^18^F]*m*-NO_2_PhSF_5_.

Given these results, we recognize that JMP© and similar professional software for statistics would be the best choice whereas a professional data analyst is available in the research team. However, we anticipate that using AI would be the preferred approach by chemists willing to analyze large datasets, given their immediacy and ease of use. While we have used Julius©, other AI tools are starting to be developed for data analysis (*e.g.* Zebra BI, Quadratic), and they differ in terms of user interface, data handling approach and functionalities. Ultimately, the choice on which tool to use depends on the task, on personal preferences and on accepted cost.

### Finding of additional radioactive products

**Fig. 9:**
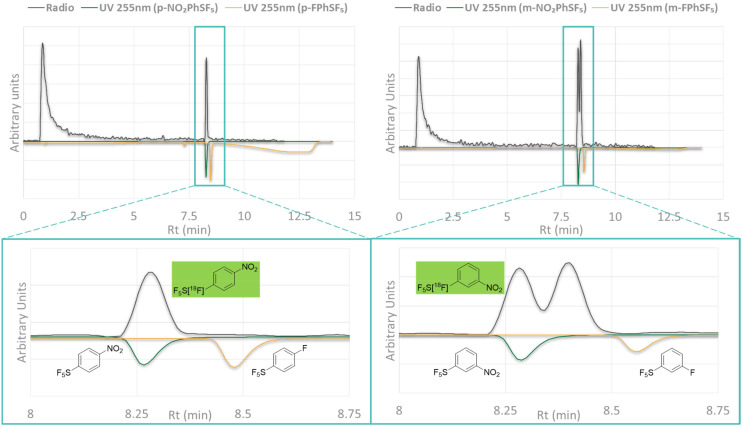
Comparison of HPLC profiles between radiolabelling mixtures (radio, tan line) and non-radioactive standards (inverted, UV@255 nm) of *m*/*p*-NO_2_PhSF_5_ (blue line) and *m*/*p*-FPhSF_5_ (yellow line).

During the 2^nd^ campaign of experiments, we noted an unexpected result from the radioactive profile, that was recorded using a brand-new Phenomenex Onyx monolith column. While we still noticed a broad peak for the [^18^F]*p*-NO_2_PhSF_5_ as in the previous analytical system, we obtained a dual radiopeak in all the radiolabelling mixtures for [^18^F]*m*-NO_2_PhSF_5_ (Fig. 9). This surprising finding gave us pause to reconsider the identity of the radioactive peaks, particularly suspecting that a NO_2_/^18^F nucleophilic aromatic substitution was instead taking place, leading to a [^18^F]*m*/*p*-FPhSF_5_.

In our system, we estimated a UV-radio time gap of 0.015 min (∼1 s) at the 2 mL min^−1^ flow rate employed in the analysis; averaging the data from 3 randomly chosen runs, we were able to confirm the identity of the [^18^F]*p*-NO_2_PhSF_5_ (time gap from UV standard: 0.012 min). On the other hand, we recorded a UV-radio time gap of 0.004 min between the 1^st^ radiopeak and the non-radioactive *m*-NO_2_PhSF_5_, thus evidencing a likely attribution to the desired product, but a time gap of 0.2 min (∼12 s) between the 2^nd^ radiopeak and the non-radioactive *m*-FPhSF_5_ standard (Fig. 9). This negative identification prompted us to formulate an alternative hypothesis for the identity of this peak, which was indeed detected in all the successful radiolabelling performed on the *m*-isomer.

### Computational analysis of potential radiolabelled isoforms

**Fig. 10:**
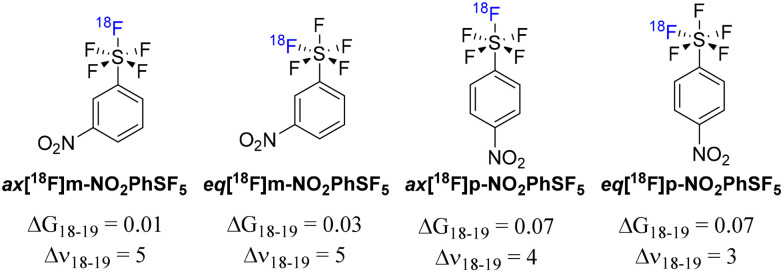
Computed Gibbs free energy and vibrational wavenumber differences for the indicated radioisotopologues; free energies are reported in kcal mol^−1^, and vibrational wavenumbers in cm^−1^. Differences were calculated by subtracting the values of the radiolabelled molecule from those of the non-radioactive precursor.

We adopted DFT modeling to try elucidating the different *R*_t_ observed in radio-HPLC traces between the ^18^F isotopically substituted NO_2_PhSF_5_ species and the corresponding stable precursor. Specifically, both the *m*-NO_2_PhSF_5_ and the *p*-NO_2_PhSF_5_ species were initially simulated without any isotopic substitution, and then with an ^18^F atom either in the axial (ax) or the equatorial (eq) positions of the SF_5_ group (Fig. 10).

Upon geometry optimization, we performed a frequency analysis to derive the isotopic substitution free energies (Δ*G*_18–19_) following the computational approach described in SI15. All Δ*G*_18–19_ values were calculated by subtracting the Gibbs free energy of the ^18^F-substituted molecule from that of the corresponding ^19^F analogue, and these values (Fig. 10) are in the same range as for similar studies conducted on P–^18^F bonds.^38^ In all the cases, the isotopically substituted species resulted in having slightly higher free energy (*i.e.* less stable) than the one containing only ^19^F. However, both eq[^18^F]*m*-NO_2_PhSF_5_ and ax[^18^F]*m*-NO_2_PhSF_5_ featured a smaller difference when compared to the *p*-NO_2_PhSF_5_ cases, suggesting a stronger stabilization of the S–^18^F bond due to the *meta* nitro group. Consequently, the *p*-NO_2_PhSF_5_ isotopologues show less favorable isotopic substitution free energies, supporting the lower RCC obtained on this substrate.

From the vibrational frequency analysis, we observed a small blueshift in the S–^18^F bond stretching vibrational mode compared to the fully ^19^F species (*i.e.* Δ*ν* in Fig. 10, calculated by subtracting the vibrational wavenumber of the S–^19^F from that of S–^18^F). This blueshift arises from the presence of a lighter ^18^F isotope, which typically correlates with a longer S–F bond. This relationship is also supported by literature on various S isotopes bonded to ^19^F.^39–43^ The largest difference in wavenumber, 5 cm^−1^, has been obtained for both the [^18^F]*m*-NO_2_PhSF_5_ isotopologues, which corresponds to a bond length change of approximately 5 mÅ, based on the derivation reported in SI16. By a similar calculation, the ^18^F substituted *p*-NO_2_PhSF_5_ isotopologues feature smaller differences, suggesting a closer structural similarity with the non-radioactive precursor. Therefore, the trends evidenced by these calculations support the lower RCC obtained with *p*-NO_2_PhSF_5_ compared to the *m*-substituted analogue (*e.g.* Gibbs free energy differences) and suggest that a different interaction of eq[^18^F]*m*-NO_2_PhSF_5_ and ax[^18^F]*m*-NO_2_PhSF_5_ with the HPLC stationary phase (more evident than with the *p*-substituted analogue) can be due to slight differences in the structures (*e.g.* vibrational wavenumber) and symmetry of each respective radiolabeled SF_5_ group, that likely dominates the chromatographic interaction given its large size and lipophilicity. It is worth noting that chromatographic differences of deuterated isotopologues are well reported in literature.^44^

Our preliminary computational attempts to justify the mechanism of substitution are presented in SI17–18, but would require robust experimental backing, which is outside the scope of this work.

Additionally to confirming by chromatography the lack of formation of *m*-FPhSF_5_, we conducted non-radioactive fluorination of *m*-NO_2_PhSF_5_ in conditions analogous to the radioactive case, to determine the possibility of direct ring fluorination at one of the 4 hydrogens, as well as potential –SF_5_ or NO_2_ substitution. Both LC-MS and ^19^F-NMR analyses did not reveal the appearance of any additional fluorinated product, thus excluding these potential reaction paths (SI19–21).

## Experimental

### Radiochemistry

#### Materials

All the reagents used were purchased from Sigma-Aldrich and used without purification unless otherwise stated. All solvents used for chromatography were purchased from Merck and were of HPLC grade. MP1 cartridges were purchased from MedChem Imaging (USA). SF_5_-containing model substrates were purchased either from Sigma-Aldrich or Oakwood Chemical (USA); amino acid analogues were synthesized following literature procedures without modification and were >95% pure by HPLC.^23–25^

#### Microfluidics reactions

Aqueous [^18^F]fluoride (5–20 GBq) was generated from an IBA cyclone 18 twin cyclotron by the ^18^O(p,n)^18^F nuclear reaction, or purchased from Cyclotek (Sydney, AUS). Radiofluoride complex was prepared in the Concentrator module of a NanoTek system (Advion, USA) using an automated process similar to what was reported in the literature (SI1).^45^ Briefly, a MP1 cartridge was used to trap the [^18^F]fluoride and was eluted dropwise with 800 μL of 0.075 M tetraethylammonium bicarbonate (TEAB, 90% CH_3_CN/10% H_2_O), evaporated to dryness and further dried by dropwise addition of 500 μL of CH_3_CN. The dried [^18^F]TEAF complex and precursors’ solution (20 mg mL^−1^ in DMSO) were pre-loaded onto two separate storage loops (P1: precursor, P3: radiofluoride). Aliquots of these solutions (10–50 μL) were delivered at a pre-determined flowrates into a microreactor (2 m × 100 μm, 15.6 μL, coiled fused silica) heated at the required temperature (±1 °C). After the reaction mixture passed though the microreactor, the total volume of the microfluidic system (∼400 μL) was swept with DMSO at a transfer rate of 100 μL min^−1^, and the mixture collected was analyzed by radio-HPLC.

#### Vial reactions

Dried [^18^F]TEAF complex was either obtained from the NanoTek automated process or manually prepared. In the latter case, [^18^F]fluoride was trapped onto a preconditioned QMA, and eluted with a solution of TEAB (5 mg) in 1 : 1 CH_3_CN/H_2_0 (2 mL), that was azeotropically dried and reconstituted in DMSO (4 mL). 100 μL of this radiolabelling stock solution was then added to 1 mL DMSO solution of precursors; the mixture was heated for the predetermined time and analyzed afterwards.

#### Radio-HPLC analysis

A Shimadzu system was used for all the HPLC analysis, and comprised of a CBM-20 controller, LC-20AD pump, SIL-20AHT autoinjector SPD-M20A PDA, Lablogic Posi-RAM gamma detector, and stationary phase Chromolith® RP-18 (Merck, 50 × 4.6 mm, 1^st^ campaign) or Onyx C18 (Phenomenex, 50 × 4.6 mm, 2^nd^ campaign). PDA spectra were recorded, using a UV target wavelength of 255 nm. Eluent used were 0.05% TFA in CH_3_CN (A) and 0.05% TFA in H_2_O (B), and a gradient elution method, at total flow of 2 mL min^−1^, was used across the whole project, holding 95% of B for 2 minutes, ramping up to 95% of A in 10 minutes, and going back to 95% of B quickly, finally holding these conditions for additional 5 minutes.

### Optimization and statistical approach

#### DoE settings

We used JMP© to create a definitive screening design and indicated the following parameters and boundaries:

Variable (discrete) parameters:

• Temperature: 30, 50, 70, 90 °C

• Flow rate P3: 10, 20, 30, 40, 50 μL min^−1^

• Flow rate ratio P1/P3: 0.5, 1, 1.5, 2

Fixed parameters:

• DMSO as solvent

• [^18^F]Et4NF as fluorinating species

• Realize design within 10 runs

The runs indicated were used for all the substrates, and are indicated in SI3.

#### BOXVIA settings

##### 1^st^ BO

The results from the respective DoE (*i.e.* comprising the obtained %RCC) were imported as training the set in the application as .csv file. The parameters to modify in the User Interface (UI) were indicated as:

• T: 30–90 °C (discrete steps of 10)

• Flow rate P3: 10–50 μL min^−1^ (discrete steps of 5)

• P1/P3 ratio: fixed to 1

The BO algorithm parameters were set at:

• Batch of 5

• EI type

• Jitter: 0.5

• Kernel: RBF

• Options: maximize, avoid re-evaluation

The experiments were then executed, and RCC was assayed by HPLC, before being added into the application window.

##### 2^nd^ BO

The RCC results were added to the original DoE training set and the same ranges were used for varying the experimental parameters. Algorithm parameters were set the same as 1^st^ BO set, except for the number of runs (batch of 3) and Jitter (0.01).

#### Statistical tools adopted

The consolidated table of results for both *p*- and *m*-NO_2_PhSF_5_ was collected in an Excel spreadsheet (SI5). The Statistical toolbox was used to calculate Pearson correlation factors and Covariance factors with RCC.

Similarly, with Excel, the same data were transferred into JMP© and the columns of data were classified for their nature (*e.g.* continuous numerical, categorical). Indicating RCC as the output parameter, the tools used in this software were: multivariate analysis, principal component analysis, predictor screening, response screening, fit least squares analysis, and neural network response predictor.

The same Excel spreadsheet file was also loaded into the User Interface of Julius© AI, and questions about their statistical analysis were asked in natural language. To respond to these questions, the AI engine created Python code that was autonomously debugged and run, providing both textual and graphical replies. Continuous interaction with the user was required to ask follow-up questions, as well as requiring to check results that seemed inconclusive, unreliable or inaccurate. The full session was then recorded and is reported in SI14.

### Computational method

All DFT calculations were carried out using ORCA 5.0.1 with the RIJCOSX approximation to reduce computational cost. The ωB97X-D3BJ functional, incorporating D3 dispersion corrections with Becke–Johnson damping, was used consistently with the def2-TZVPP basis set. Fluorine mass was modified in frequency calculations to simulate ^18^F isotope effects at axial and equatorial positions. Solvent effects from a 50 : 50 water/acetonitrile mix were modelled using CPCM with a dielectric constant of 47.07 and refractive index of 1.3456.

Thermal contributions to the Gibbs free energy were calculated as:*G* = *E* + ZPE + *H*(298.15 K) − 298.15 × *S*(298.15 K).

We simulated both *m*- and *p*-NO_2_PhSF_5_, with and without ^18^F at axial or equatorial positions. Geometry optimizations and frequency analyses were performed to calculate the isotopic substitution free energies (Δ*G*_18–19_):Δ*G*_18–19_ = *G*(^18^F) − *G*(^19^F)

Additional calculation details are provided in SI16.

## Conclusions

This work demonstrates a novel radiofluorination reaction involving the isotopic exchange of aromatic pentafluorosulfanyl moiety. We successfully applied this process to nitro-substituted aromatics, and believe other aromatic structures could also undergo such a labelling, as demonstrated by the Tyr analogue. Interestingly, this exchange is likely not a conventional nucleophilic substitution, as best RCCs are obtained at mild temperatures, whereas the high temperatures (*e.g.* >90 °C) conventionally used in radiofluorinations reduced conversion. We confirmed this peculiar trend by performing experiments using a novel strategy for unbiased optimization, and analyzing the results with conventional statistical methods and innovative Artificial Intelligence tools. We serendipitously discovered that the HPLC analysis of the radiolabelling of *m*-NO_2_PhSF_5_ resulted in the formation of two radioactive peaks, that we propose being positional radioisotopologues, namely eq[^18^F]*m*-NO_2_PhSF_5_ and ax[^18^F]*m*-NO_2_PhSF_5_. These isomers may interact differently with the HPLC stationary phase, potentially providing the first case of differential behaviour between ^19^F and ^18^F. A similar phenomenon frequently occurs with deuterated compounds^44^ and has never been observed or proposed for fluorine isotopes. Our claim can be supported by computation trends for alternative structures, that shows how these positional differences are more evident in the *m*-NO_2_ case than the *p*-NO_2_ analogue, which did not display such HPLC peak splitting pattern. While practically limited to nitroaromatics, we believe our discovery paves the way for incorporating a new radiofluorinated fragment into the molecular design of radiopharmaceuticals. However, further investigations are required to fully comprehend the scope of the reaction and the exchange mechanism. Additionally, a direct experimental evidence demonstrating the proposed formation of positional isotopologues is needed; such proof could involve the non-carrier-added labelling of typically unstable Ar-SF_4_Cl,^5,6^ that, in a few cases, can be isolated and reveal the axial position of the chlorine atom.^15^

## Author contributions

G. P., H. H., L. H. and A. U. conceptualized the project; H. H., G. W., J. H., P. R., R. J. performed the experiments; G. P., L. H., A. U., A. B. and P. S. supervised the experimental work; W. H., F. C. and M. L. managed the computational aspects. G. P. wrote the draft, that was reviewed by all the authors.

## Conflicts of interest

There are no conflicts to declare.

## Supplementary Material

OB-023-D5OB01419K-s001

## Data Availability

All pertinent data are reported in the publication and the supporting information (SI). Supplementary information is available. See DOI: https://doi.org/10.1039/d5ob01419k.
